# Think twice before you tell

**DOI:** 10.1007/s12471-014-0536-x

**Published:** 2014-03-04

**Authors:** A. A. M. Wilde

**Affiliations:** Academic Medical Centre Amsterdam, Amsterdam, the Netherlands

Answer to the Rhythm Puzzle

At first glance the ECG looks very abnormal. The P-wave morphology is not compatible with sinus rhythm, the conduction intervals are normal, and the QRS complexes are very abnormal in amplitude (micro-voltages in the extremity leads). In lead II there is almost no signal at all.

**Fig. 2:**
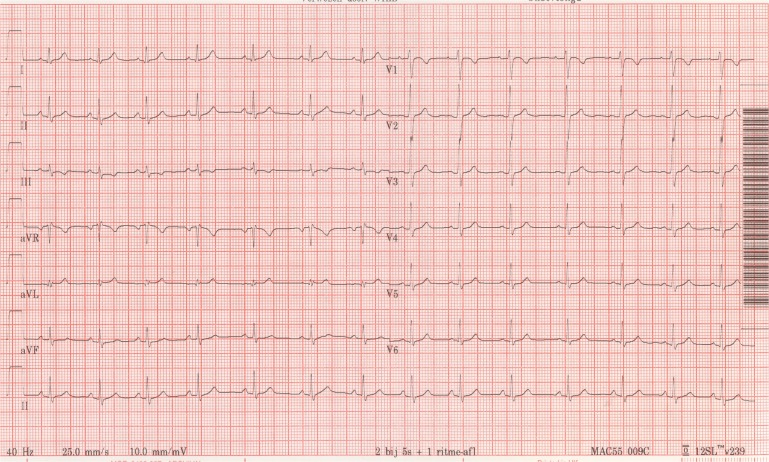
Second ECG (with correct positions)

Although micro-voltages in the extremity leads are a common feature in one of the most prevalent familial cardiomyopathies in the Netherlands [1], the abnormal P-wave configuration and the very abnormal signal in lead II should raise the possibility of aberrant placing of the extremity leads. Also the normal echo is to some extent reassuring and raises the possibility of a technically incorrect ECG. A switch of the left and right arm would explain the negative P-wave in the lateral leads and a switch of the right arm and the right leg explains the virtual absence of signals in lead II (and AVF). A new ECG was taken and that proved to be completely normal (Fig. 2). So, you had better think twice before you tell the patient that she has to worry.
